# Muscle wasting and the temporal gene expression pattern in a novel rat intensive care unit model

**DOI:** 10.1186/1471-2164-12-602

**Published:** 2011-12-13

**Authors:** Monica Llano-Diez, Ann-Marie Gustafson, Carl Olsson, Hanna Goransson, Lars Larsson

**Affiliations:** 1 Department of Clinical Neurophysiology, Uppsala University, Uppsala, Sweden; 2 Department of Medical Sciences, Uppsala University Hospital, Uppsala, Sweden

## Abstract

**Background:**

Acute quadriplegic myopathy (AQM) or critical illness myopathy (CIM) is frequently observed in intensive care unit (ICU) patients. To elucidate duration-dependent effects of the ICU intervention on molecular and functional networks that control the muscle wasting and weakness associated with AQM, a gene expression profile was analyzed at time points varying from 6 hours to 14 days in a unique experimental rat model mimicking ICU conditions, i.e., post-synaptically paralyzed, mechanically ventilated and extensively monitored animals.

**Results:**

During the observation period, 1583 genes were significantly up- or down-regulated by factors of two or greater. A significant temporal gene expression pattern was constructed at short (6 h-4 days), intermediate (5-8 days) and long (9-14 days) durations. A striking early and maintained up-regulation (6 h-14d) of muscle atrogenes (muscle ring-finger 1/tripartite motif-containing 63 and F-box protein 32/atrogin-1) was observed, followed by an up-regulation of the proteolytic systems at intermediate and long durations (5-14d). Oxidative stress response genes and genes that take part in amino acid catabolism, cell cycle arrest, apoptosis, muscle development, and protein synthesis together with myogenic factors were significantly up-regulated from 5 to 14 days. At 9-14 d, genes involved in immune response and the caspase cascade were up-regulated. At 5-14d, genes related to contractile (myosin heavy chain and myosin binding protein C), regulatory (troponin, tropomyosin), developmental, caveolin-3, extracellular matrix, glycolysis/gluconeogenesis, cytoskeleton/sarcomere regulation and mitochondrial proteins were down-regulated. An activation of genes related to muscle growth and new muscle fiber formation (increase of myogenic factors and JunB and down-regulation of myostatin) and up-regulation of genes that code protein synthesis and translation factors were found from 5 to 14 days.

**Conclusions:**

Novel temporal patterns of gene expression have been uncovered, suggesting a unique, coordinated and highly complex mechanism underlying the muscle wasting associated with AQM in ICU patients and providing new target genes and avenues for intervention studies.

## Background

All critically ICU patients suffer from severe wasting and impaired muscle function, which delay respirator weaning and persist long after hospital discharge; thus reducing quality of life [1,2]. Although muscle wasting in ICU patients may be related to the primary disease, it also devolves from the interventions used in modern anaesthesiology and intensive care: Prolonged mechanical ventilation, post-synaptic neuromuscular transmission blockade (NMB), sedation, and systemic corticosteroid hormone treatment have all been proposed as factors triggering the severe muscle wasting, paralysis, impaired respiratory function, and partial or complete loss of the motor protein myosin in ICU patients who develop Acute Quadriplegic Myopathy (AQM). Sepsis, organ transplantation, multi-organ failure, and hyperglycemia are also hypothesized risk factors for AQM [3-6]. We have recently demonstrated that complete mechanical silencing, i.e., absence of weight bearing and internal strain in the muscle caused by muscle contraction, induces a phenotype which closely resembles that of AQM in ICU patients [7]. The myosin loss and muscle wasting follows a temporal sequence with an initial sparing of both muscle function, mass and myosin content followed by a progressive loss of muscle force that exceeds the loss in muscle mass due to a preferential loss of the motor protein myosin [7-9].

Acute quadriplegic myopathy, also known as critical illness myopathy (CIM), thick filament myosin myopathy, acute myopathy in severe asthma and myopathy of intensive care [3], was for many years considered to be rare and of limited clinical significance, but in the past two decades the number of reported cases with AQM has substantially increased. Recent studies show that approximately 50% of ICU patients with sepsis, multi-organ failure or prolonged mechanical ventilation present significant neuromuscular dysfunction [10]. This muscle wasting and weakness may persist 5 years after hospital discharge, drastically impairing quality of life of survivors as well as increasing morbidity and financial costs [1,11,12].

There is a strong interest in the fundamental molecular mechanism of muscle atrophy, including the complex and highly ordered mechanisms of protein synthesis and degradation, the suppression of mitochondrial related bioenergetic pathways, cell proliferation and differentiation, and oxidative stress [13,14]. In AQM, muscle wasting involves the activation of three proteolytic systems: ubiquitin-proteasome, autophagy-lysosome, and the calcium-dependent calpains, as well as inactivation of specific Na channels, activation of the TGF-β/MAPK cascade, and apoptotic pathways [15,16]; however, how and when these mechanisms are activated remain poorly understood.

There are several independent factors that complicate the study of mechanisms underlying the muscle wasting and loss of muscle function in ICU patients with AQM, such as differences in primary disease, different pharmacological treatments, exposure to different causative agents, and delay of muscle biopsies until several weeks after ICU admission. To effectively unravel underlying mechanisms, experimental animal models mimicking the ICU intervention are needed. The most common animal model used to date is a rat model with unilateral peripheral denervation of one hind limb combined with high levels of systemic corticosteroid administration [17,18]; other models of disuse and muscle unloading are hind limb suspension, spaceflight, joint immobilization, and spinal cord isolation [19-22]. All these experimental models induce muscle atrophy, but lack significant components of muscle wasting seen in ICU patients due to deep sedation or NMB, such as long-term mechanical ventilation and mechanical silencing caused by lack of weight bearing, and the internal strain produced by activation of contractile elements. There is accordingly a strong demand of an experimental ICU model mimicking ICU conditions, i.e., key factors essential for the muscle wasting and paralysis in ICU patients who develop AQM [23]. We have previously used a porcine ICU model, in which piglets are mechanically ventilated and exposed to NMB, corticosteroids and/or sepsis for 5 days [24]. This model has given us valuable information regarding the effects of these different triggering factors separately or in combination during the early phase of the disease [8]; but, this model also has disadvantages such as high cost, and logistic problems that limit the duration of the experiments and the study of proteins with a slow turnover rate such as contractile proteins.

In order to improve our understanding of duration-dependent effects of the ICU intervention on molecular and functional networks that control the muscle wasting and weakness, we have used a unique experimental rat model that mimics the ICU condition. This model includes key elements such as mechanical ventilation, mechanical silencing, NMB and extensive monitoring for durations varying from 6 hours to 14 days, without the confounding influences of differences in systemic disease and pharmacological treatment. Gene array analyses with this model reveal that, in response to the ICU intervention, there is a complex, unique, and highly coordinated activation of protein synthesis, degradation, protective mechanisms, and intracellular signalling.

## Results

Out of 26,209 probe sets on the array, 1583 were significantly up- or down-regulated in at least one of the 0.25-4, 5-8 and 9-14 day groups compared with the control group. 128, 1182 and 1115 gene probes passed the cut-off criteria (minimum ± 2 fold change with adjusted *p*-value < 0.05) in the 0.25-4, 5-8 and 9-14d groups, respectively. The dramatic increase of differentially expressed genes in the 5-8d and 9-14d groups compared with the 0.25-4d group agrees with the marked gastrocnemius muscle loss, i.e., 40% in the 5-8d and 51% in the 9-14d groups compared with the modest 11% muscle mass loss in the 0.25-4d group.

**Figure 1:**
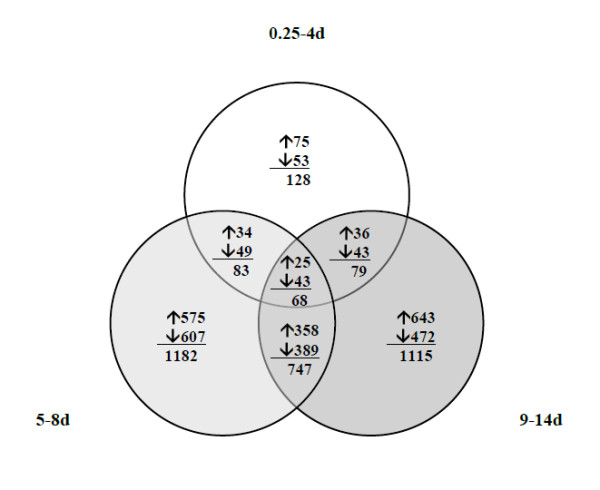
**Number of genes differentially expressed at short (0.25-4d), intermediate (5-8d) and long (9-14d) durations**. Number of genes regulated at more than one time point are found in overlapping regions. Upward arrows indicate up-regulation and downward arrows indicate down-regulation.

The Venn diagram shows overlapping genes at the three durations (Figure 1). Only 68 differentially expressed genes were common in all three groups. Further, there was little overlap between the pairs 0.25-4d - 5-8d and 0.25-4d - 9-14d groups (83 and 79 genes respectively), while 747 genes overlapped between the 5-8 and 9-14d groups, indicating that these two groups shared almost half of the 1583 significantly regulated genes.

### *K*-means cluster analysis

**Figure 2:**
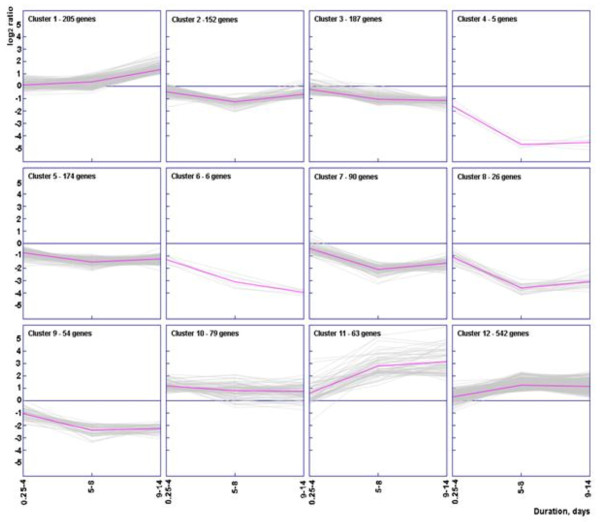
**Results from *K*-means cluster analysis**. Genes were divided into 12 clusters using a *K*-means cluster analysis algorithm, which distinguishes sets of genes with similar expression profiles with respect to time. Relative expression levels (log_2 _ratio) for each gene and the average expression values (magenta line) are shown at different time points (0.25-4d, 5-8d and 9-14d) compared to control conditions.

Results of *K-*means clustering analysis, separating the 1583 differentially expressed genes into 12 clusters, allowed the reconstruction of different temporal expression patterns (Figure 2). Each data point represents the average expression (log_2 _ratio) value at different time points (0.25-4d, 5-8d and 9-14d).

Clusters 1, 10, 11 and 12 contained up-regulated genes. Cluster 10 had 79 genes with an early (0.25-4d) and sustained response (5-8d) of 2-fold change (log_2_1), decreasing slightly at the last time point (9-14d). Atrogenes (muscle ring-finger 1/tripartite motif-containing 63 (*Murf1/Trim63*) and F-box protein 32/atrogin-1 (*Fbox32/atrogin-1*)) and their regulator, forkhead box O1 (*Foxo1)*, were in this cluster. Cluster 12 had by far the largest number of genes (542) with a maintained up-regulation around 2-fold (log_2_1) from 5 to 14 days. These genes are involved in proteolysis (ubiquitin-proteasome, calpain and autophagy-lysosome systems), apoptosis, DNA repair, protein synthesis, endoplasmic reticulum-associated degradation (ERAD), antioxidant and oxidative stress response genes, amino acid regulation and chaperone activity. Cluster 11 had 63 genes up-regulated from 5 to 14 days, but unlike cluster 12, they were highly expressed with an average 8-fold change (log_2_3). These genes belong to muscle development, cell cycle arrest, metallothioneins and autophagy-lysosome system. Cluster 1 contained 205 genes which were only up-regulated at the longest duration (9-14d), i.e., genes mainly involved in immune response and apoptosis.

The remaining clusters represented different expression patterns of down-regulated genes during the time course. Clusters 4, 5, 6, 8 and 9 presented an early significant decline in expression response (0.25-4d) that was maintained at the longer durations (from 5 to 14 days). Clusters 6 and 8 included 6 and 26 down-regulated genes involved in muscle contraction (the β/slow and the fast IIa myosin heavy chains (*MyHC-beta/Myh7 *and *MyHC-IIa/Myh2) *and myosin light chain 2 and 3 (*MyLC2/Myl2 *and *MyLC3/Myl3*), that were significantly down-regulated between 2- and 3-fold (log_2_ (-1) and log_2_ (-1.5)) at 0.25-4d, continued to declined more than 8-fold (log_2_ (-3)) from 5 to 14 days. Clusters 5 and 9 included 174 and 54 genes respectively, well represented by growth factors, control of cell cycle and regulation of proliferation and differentiation with a 2-fold down-regulation at 0.25-4d (log_2_ (-1)) and a moderate decline (between 2 and 4-fold change) at intermediate and long durations (5-14d). Five genes were grouped into cluster 4, but they did not share any functional characteristic. Clusters 2, 3 and 7 showed a significant down-regulation of gene expression at the two longest durations (5-14d). Cluster 7 included 90 down-regulated genes showing around 4-fold change (log_2_ (-2)), mainly genes involved in developmental processes and genes coding some sarcomeric and extracellular matrix proteins. Clusters 2 and 3 included 152 and 187 genes respectively, down-regulated 2-fold, i.e., glycolysis/gluconeogenesis, respiratory chain, tricarboxylic acid (TCA) cycle, extracellular matrix structure, cell adhesion, developmental processes and cytoskeleton/sarcomere genes.

In almost all clusters, there were genes of metabolism, transport, signal transduction and transcription (detailed list of the genes in each cluster with associated functional categories are listed in Additional file 1).

**Figure 3:**
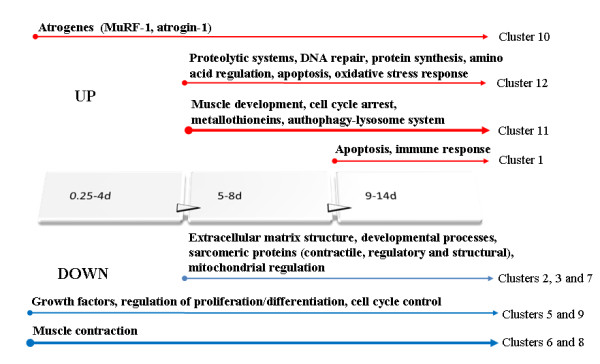
**Graphical summary of significant temporal gene expression patterns according to *K*-means cluster analysis**. Red and blue arrows show up- and down-regulation, respectively. Thin arrows indicate 2- to 4-fold change and thick arrows more than 4-fold change of gene expression.

According to *K*-means clustering results, a graphical summary of significant temporal gene expression patterns at the different time points are shown in Figure 3.

### Gene functional classification independent of *K*-means clustering

In order to obtain more detailed temporal expression patterns of individual genes belonging to the same functional categories, genes were grouped together according to their function independent of *K*-means clustering.

#### Ubiquitin-proteasome system (UPS)

Most Ub-proteasome genes were up-regulated 2-3-fold at 5-8d, followed by a slight decrease at 9-14d. The two muscle-specific E3 ligases, *atrogin-1/Fbox32 *and *Murf1/Trim63 *showed an early (0.25-4d) and sustained (5-14d) 2-3-fold up-regulation.

#### Autophagy-lysosome system

The lysosomal proteases, cathepsins (*Ctsl1, Ctss *and *Lgmn*) were up-regulated at different time points. *Ctsl1 *showed an early up-regulation (0.25-4d) and increased to more than 10-fold from 5 to 14 days while legumain (*Lgmn*) and *Ctss *showed a later and lower (2-3-fold) expression response. The microtubule-associated protein 1 light chain 3 beta (*Map1lc3b*), playing an essential role for formation of authophagosomes, was up-regulated 2-3-fold during the whole course of the intervention. Runt related transcription factor 1 (*Runx1*) and cathepsin inhibitors (cystatins (*Cst*)) showed a more than 10-fold up-regulation from 5 to 14 days.

#### Calpain system

Calpains, nonlysosomal calcium-dependent proteases, were regulated from 5 to 14 days. Calpain 1 (*Capn1*) did not change significantly, however, calpain 2 (*Capn2*) was up-regulated ~2-fold while the muscle-specific isoform, calpain 3 (*Capn3*), was down-regulated (5-14d).

#### Endoplasmic reticulum-associated degradation (ERAD)

ER degradation enhancer, mannosidase alpha-like 1 and 2 (*Edem1, Edem2*), ER lipid raft associated 1 (*Erlin1*), UBX domain protein 4 (*Ubx4*) and Der1-like domain family member 2 were up-regulated ~2-fold from 5 to 14 days.

#### Sarcomeric proteins

These were down-regulated mainly from 5 to 14 days with the exception of the up-regulation of myosin binding protein H (*Mybph*). Genes coding the β/slow and the fast IIa MyHC isoforms (*Myh7 and Myh2*) and regulatory (*Myl2*) and essential (*Myl3*) myosin light chains showed a 2-3-fold down-regulation from 6 h to 4 days, but did not pass the cut-off adjusted *p*-value <0.05. From 5 to 14 days, *Myh7*, *Myh2*, *Myl2 *and *Myl3 *were dramatically down-regulated, i.e., 5-, 11-, 10-, and 6-fold (5-8d) and 13-, 8-, 13- and 17-fold (9-14d), respectively. Other sarcomeric proteins such as the contractile proteins alpha cardiac myosin heavy chain 6 (*Myh6*), myosin light chain kinase 2 (*Mylk2*) and myosin light chain 1 (*Myl1*), the actin associated regulatory proteins tropomyosin (*Tpm3*) and tropomodulin (*Tmod4*) and structural sarcomeric proteins, such as LIM domain, actinin, myomesin and myozenin, were also down-regulated from 5 to 14 days.

#### Extracellular matrix

Most extracellular matrix genes such as members of collagen family, laminins (*Lamb2, Lama2*), fibulins and microfibrillar associated proteins (*Mfap4, Mfap3l, Mfap5*) among other proteins were down-regulated from 5 to 14 days.

#### Protein synthesis

Eukaryotic translation initiation and elongation factors were up-regulated 2-3-fold from 5 to 14 days as most ribosomal proteins. The protein synthesis repressor, eukaryotic translation initiation factor 4E binding protein 1 (*Eif4ebp1*) was up-regulated from 6h to 8 days and followed by a dramatic decline from 9 to 14 days.

#### Oxidative stress response

There was a global activation of antioxidant and chemoprotective genes during the course of the ICU intervention. Heme oxygenase 1 (*Hmox*), aldehyde oxidase 1 (*Aox1*), beta polypeptide xanthine dehydrogenase (*Xdh*) were up-regulated early (6h-4d) and sustained (5-14d) while NAD(P)H dehydrogenase quinone 1 (*Nqo1*), biliverdin reductase B (*Blvre*), aldehyde dehydrogenase 3 family member A2 (*Alh3a2*) and several enzymes of the glutathione *S*-transferase (GST) family were up-regulated from 5 to 14 days. The metallothioneins 2a and 1 (*Mt2a *and *Mt1) *were up-regulated more than 8-fold at 0.25-4d, reaching more than 15-fold up-regulation at 9-14d group.

#### Cellular cycle regulators

There was a noticeable up-regulation, especially from 5 to 14 days, of genes intervening in growth arrest, i.e., growth arrest and DNA-damage-inducible alpha (*Gadd45a*),mediator of DNA damage checkpoint 1 (*Mdc1*), MAD2 mitotic arrest deficient-like 2 (yeast) (*Mad2l2*), block of proliferation 1 (*Bod1*), genetic suppressor element 1 (*Gse1*) and the growth inhibitors, cyclin-dependent kinase inhibitor 2b, p15 (*Cdknb2*) and cyclin-dependent kinase inhibitor 1a, p21 (*Cdkna1*).

The myelocytomatosis oncogene (*Myc*), stimulating muscle growth-related genes and apoptosis, was up-regulated 4-fold at all durations (0.25-14d).

#### Apoptosis

A late up-regulation was observed in the caspase cascade at 9-14d, i.e., caspase-4 (*Casp4*) was up-regulated 2.5-fold and caspases 3, 1 and 12 (*Casp3*, *Casp1 *and *Casp12*) did not pass ± 2-fold change cut-off, but its expression was 1.7-fold higher. Other pro-apoptotic genes such as programmed cell death 6 (*Pdcd6*), SIVA1 apoptosis-inducing factor (*Siva1*), BH3 interacting domain death agonist (*Bid*), cell death-inducing DFFA-like effector a (*Cidea*) and apoptosis antagonizing transcription factor (*Aatf*) increased their expression from 5 to 14 days. In parallel with the activation of apoptosis, the apoptotic repressor, nucleolar protein 3 (*Nol3*) and the anti-apoptotic gene B-cell leukemia/lymphoma 2 related protein A1d (*Bcl2a1d*) were up-regulated 2-fold at the longest duration (9-14d).

#### Heat shock proteins/chaperones

Heat shock proteins *Hsp90aa1*, *Hspb8*, cyclophilin A (*Ppia*), and some members of DnaJ (*Hsp40*) family were up-regulated 2-3-fold from 5 to 14 days. Heat shock 105kDa/110kDa protein (*Hsph1*) and translocase of outer mitochondrial membrane 34 (*Tomm34*) increased 2-fold after 9 days. However, the muscle atrophy protective heat shock protein, alpha-crystallin-related B6 (*Hspb6*) and other chaperones were down-regulated from 5 to 14 days.

#### Muscle development

Myogenin (*Myog*) was up-regulated 2-fold at 0.25-4 days, followed by a 13-fold increase (5-14d) and the myogenic factors, *Myf5 *and *Myf6*, were up-regulated 3-4-fold from 5 to 14 days. The inhibitor of DNA binding 2 (*Id2*) was up-regulated 2-fold at the longest duration. The skeletal muscle receptor tyrosine kinase (*Musk*) was gradually up-regulated during the time course of the ICU intervention reaching 2.2-, 5.4- and 9.3-fold increase at short, intermediate and long durations, respectively.

Histone deacetylase 4 (*Hdac4*) that represses the muscle transcription factor myocyte enhancer factor 2 (*Mef2*), being a contributor of muscle dysfunction [25], increased around 3-fold at the 3 time points (0.25-14d).

Some genes implicated in muscle development and growth also showed a significant down-regulation, such as fibroblast growth factors 1, 2 and 6 (*Fgf1*, *Fgf2*, *Fgf6*), supervillin (*Svil*) invoved in myosin II assembly and focal adhesion and junctophilin (*Jph1*) were all down-regulated at the intermediate and longest durations (5-14d). Tripartife motif-containing 72 (*Trim7*2), a muscle-specific protein that plays a central role in cell membrane repair, and vestigial like 2 (Drosophila) (*Vgll2*), a cofactor of *Mef2 *and *Tef1 *required for skeletal muscle differentiation, were down-regulated from 5 to 14 days. Kyphoscoliosis peptidase (*Ky*), a cytoskeleton-associated protease essential for normal muscle growth, maturation and stabilization of neuromuscular junction and required for a hypertrophic response in muscle [26], was down-regulated 4-fold (0.25-4d), 30-fold (5-8d) and 15-fold (9-14d).

Embigin homolog mouse (*Emb*) was recently identified as a nerve terminal sprouting factor at the neuromuscular junction that is up-regulated in denervated muscle [27]. Under ICU intervention, *Emb *was strongly up-regulated from 5 to 14 days (36- and 59-fold change at 5-8d and 9-14d, respectively.

The muscle specific caveolin-3 (*Cav3*) was significantly down-regulated 4.8-fold from 5 to 14 days.

#### Immune response

Many genes from this group, such as chemokines, antigens, immunoglobulins, complement components, and interferons, were primarily up-regulated at 9-14d.

#### Metabolism

Carbohydrates, lipids, fatty acids, aldehydes and steroids metabolic genes and genes participating in glycolysis, gluconeogenesis and mitochondria regulation (oxidative phosphorylation, citric acid cycle) were down-regulated from 5 to 14 days, while amino-acid metabolism was up-regulated during the same time period.

Complete information (fold change, adjusted *p*-values) and functional classification of genes previously mentioned and other differentially expressed genes involved in transport, cytoskeleton structure and regulation, mRNA processing, cell adhesion and genes that regulate growth, proliferation, differentiation, transcription and signalling are listed in Additional file 2.

#### Signalling pathways

List of differentially regulated genes with their corresponding fold change and adjusted *p*-value at early (0.25-4d), intermediate (5-8d) and long (9-14d) durations are shown in Table 1. The large majority of these genes were significantly switched on from 5 to 14 days.

**Table 1:** List of differentially expressed genes at least in one time point involved in signalling pathways.

Gene Name	Gene Symbol	FC (0.25-4d)	adj.-*p*	FC (5-8d)	adj.-*p*	FC (9-14d)	adj.-*p*
		**IGF1-AKT-FOXO signalling**					
v-akt murine thymoma viral oncogene homolog 3 (protein kinase B, gamma)	Akt3	1.1	7.7E-01	1.6	1.2E-02	2.5	2.9E-06
insulin-like growth factor 1 receptor	Igf1r	1.2	1.2E-01	2.0	2.6E-06	1.8	4.5E-06
insulin-like growth factor 2 mRNA binding protein 2	Igf2bp2	1.1	8.1E-01	2.2	4.8E-05	1.6	2.5E-03
forkhead box O1	Foxo1	3.1	3.1E-04	1.2	6.4E-01	-1.1	7.0E-01
insulin-like growth factor binding protein 6	Igfbp6	-1.2	7.0E-01	-4.6	1.2E-04	-2.5	4.8E-03
insulin receptor substrate 1	Irs1	-1.9	2.9E-02	-4.2	8.4E-06	-3.9	2.4E-06
		**NF-*k*B signalling**					
ectodysplasin A2 receptor	Eda2r	1.5	6.5E-01	4.7	8.5E-03	4.4	4.1E-03
nuclear factor of kappa light polypeptide gene enhancer in B-cells inhibitor, epsilon	Nfkbie	1.1	8.8E-01	2.1	6.2E-02	3.5	6.8E-04
mitogen-activated protein kinase kinase kinase 14	Map3k14	1.7	4.3E-01	3.5	3.0E-02	3.3	1.7E-02
nuclear factor of kappa light polypeptide gene enhancer in B-cells 2, p49/p100	Nfkb2	1.3	6.5E-01	3.6	5.6E-03	3.1	5.5E-03
lipopolysaccharide-induced TNF factor	Litaf	1.7	4.1E-03	3.5	1.3E-07	3.0	5.2E-08
mitogen-activated protein kinase kinase kinase kinase 4	Map4k4	1.1	8.5E-01	3.5	9.4E-05	2.5	8.2E-04
Tnf receptor-associated factor 2	Traf2	-1.0	1.0E+00	2.8	2.1E-03	2.4	2.4E-03
interleukin-1 receptor-associated kinase 4	Irak4	1.2	2.2E-01	1.6	1.6E-03	2.2	4.0E-07
activating signal cointegrator 1 complex subunit 2	Ascc2	1.4	1.0E-01	2.4	1.2E-04	2.1	2.8E-04
		**TGF-β signalling**					
TGFB-induced factor homeobox 1	Tgif1	2.5	1.7E-03	5.7	1.1E-06	5.4	1.7E-07
follistatin	Fst	1.0	9.5E-01	1.8	5.5E-02	4.1	3.5E-06
latent transforming growth factor beta binding protein 1	Ltbp1	1.2	8.0E-01	4.2	2.7E-03	4.1	9.9E-04
activin A receptor, type IIB	Acvr2b	-1.4	3.7E-02	-1.5	3.7E-03	-2.0	2.3E-06
activin A receptor, type I	Acvr1	-1.6	6.3E-03	-2.1	2.2E-04	-2.3	5.5E-06
myostatin	Mstn	1.8	1.5E-01	-5.6	5.0E-05	-3.7	2.8E-04
		**JAK-STAT signalling**					
signal transducer and activator of transcription 2	Stat2	1.2	5.4E-01	1.8	5.3E-03	2.4	1.5E-05
jun B proto-oncogene	Junb	1.9	1.7E-01	1.7	2.0E-01	2.3	1.9E-02
Janus kinase 2	Jak2	1.4	9.8E-02	1.8	5.1E-03	2.2	6.8E-05
suppressor of cytokine signaling 2	Socs2	-2.6	6.6E-06	-3.5	2.9E-07	-2.1	5.0E-05
cytokine inducible SH2-containing protein	Cish	-3.7	3.9E-02	-6.2	3.6E-03	-4.6	5.2E-03
		**p38 MAPK cascade**					
mitogen-activated protein kinase-activated protein kinase 2	Mapkapk2	-1.2	1.4E-01	-1.9	8.2E-06	-2.2	4.4E-08
mitogen-activated protein kinase kinase 6	Map2k6	-2.4	6.7E-02	-9.4	1.6E-05	-7.0	1.6E-05
dual specificity phosphatase 1	Dusp1	-2.0	1.1E-01	-4.9	3.0E-04	-3.8	5.2E-04
dual specificity phosphatase 10	Dusp10	-3.2	7.5E-05	-1.5	1.3E-01	-1.2	4.0E-01
dual specificity phosphatase 14	Dusp14	-1.3	3.2E-01	-2.2	1.9E-03	-1.7	1.5E-02
dual specificity phosphatase 8	Dusp8	-1.9	2.7E-03	-4.5	7.8E-08	-7.1	5.4E-11
		**p53 signalling**					
tumor protein p53 inducible nuclear protein 1	Tp53inp1	2.6	2.7E-05	4.5	7.7E-08	3.9	1.2E-08
nucleophosmin (nucleolar phosphoprotein B23, numatrin)	Npm1	1.4	2.4E-01	2.5	1.7E-04	2.5	5.5E-05
cytoplasmic FMR1 interacting protein 2	Cyfip2	-1.0	9.9E-01	1.1	8.4E-01	2.1	3.2E-05
Bri3 binding protein	Bri3bp	1.1	6.2E-01	2.1	1.2E-04	2.1	1.6E-05
homeodomain interacting protein kinase 2	Hipk2	1.1	2.6E-01	2.0	8.7E-08	1.7	4.8E-07
tumor protein p53 inducible nuclear protein 2	Trp53inp2	-1.3	3.6E-01	-2.4	1.1E-03	-2.2	1.1E-03

FC = fold change at the three different time points (0.25-4d, 5-8d, 9-14d) with its corresponding adjusted *p*-value.

*IGF1-AKT-FOXO signalling. Igf1r *was up-regulated around 2-fold (5-14d). *Foxo1 *was up-regulated 3-fold only in the early phase (0.25-4d), promoting the up-regulation of atrogenes in this early period. *Akt3 *was up-regulated 3.5-fold at the longest duration (9-14d).

*NF-*κ*B signalling*. TNF-α contributes to the muscle loss through activation of the NF*-*κB family. Several genes (*Traf2*, *Nfkb2*, and *Nfkbie*) and positive activators of this pathway *Eda2r*, *Irak4*, *Ascc2*, *Litaf*, *Map4k4*, and *Map3k14*) were up-regulated from 5 to 14 days.

*TGF-β signalling*. Myostatin (*Mstn*), the negative regulator of muscle growth, was up-regulated 1.8 fold, with an adjusted *p-*value higher than 0.05. However, *Mstn *was significantly down-regulated from 5 to 14 days. The Mstn inhibitor, follistatin (*Fst*) was also up-regulated in that time period (5-14d). The Mstn receptors, *Acvr2b *and *Acvr1 *were down-regulated 2-fold at 9-14d and 5-14d, respectively. Other negative regulators of this pathway such as *Tgif1*, a corepressor of *Smad2*, and *Ltbp1 *were up-regulated 4-6-fod from 5 to 14 days.

*JAK-STAT signalling*. This signalling pathway was activated as indicated by the ~2-fold up-regulation of *Jak2*, *Stat2 *and *JunB *from 9 to 14 days. The suppressors of cytokine signaling, *Cish *and *Socs2*, were down-regulated from 6 h to 14 days.

*p38 MAPK cascade*. Several genes of this pathway were down-regulated, generally after 5 days: the p38 MAPK substrate, *Mapkapk2/Mk2*, *Map2k6 *and dual specificity phosphatases (*Dusp*)

*p53 signalling*. Genes that participate in this apoptosis pathway were mainly up-regulated from 5 to 14 days.

**Figure 4:**
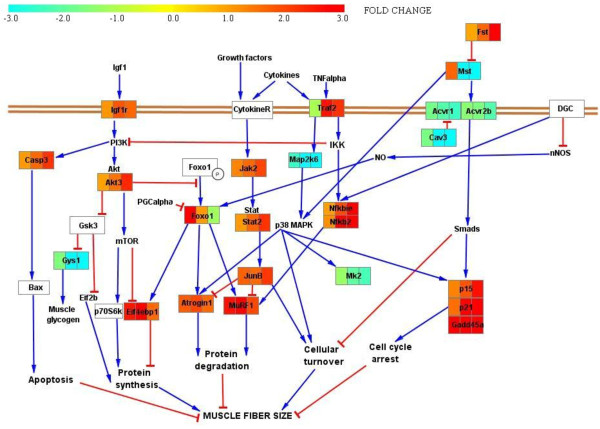
**Schematic illustration of the major pathways that control adult skeletal muscle size**. Significant differentially expressed genes playing important roles in these pathways were mapped according to their expression level at the three time point groups (0.25-4d, 5-8d and 9-14d); turquoise = down-regulated genes, red = up-regulated genes and yellow = unregulated genes. Red lines indicate inhibition and blue lines activation.

A schematic illustration of the major pathways that control adult skeletal muscle size are displayed in Figure 4, i.e., genes significantly affected by the experimental ICU intervention.

### Immunofluorescence cytochemistry results

**Figure 5:**
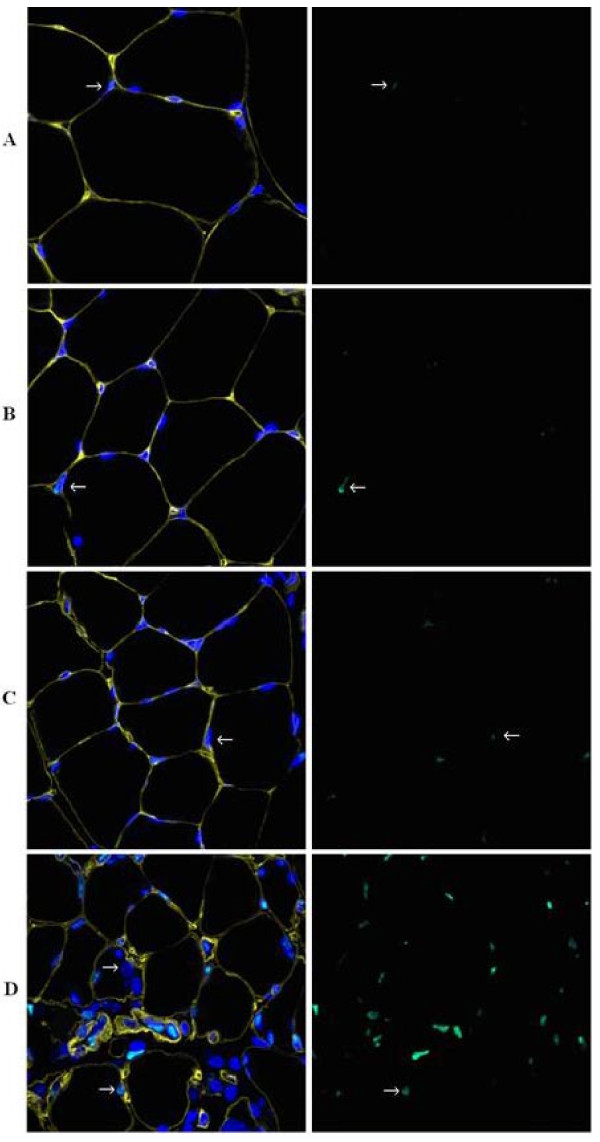
**Immunofluorescence cytochemistry images**. A: control rats, B: 0.25h-4d, C: 5-8d and D: 9-14d. Yellow shows laminin, blue is DAPI and green cleaved caspase-3. Arrows in A and C show apoptotic myonucleus, arrow in B shows apoptotic nucleus outside laminin, arrow in D shows a centralized swelled nucleus that might be an activated satellite cell.

Apoptotic nuclei were visualized by staining with cleaved caspase-3 antibody and 4´,6-diamidino-2-phenylindole (DAPI). When DAPI and cleaved caspase-3 colocalized, the nucleus was considered apoptotic. To determine whether the apoptotic nuclei were inside or outside the basal lamina of the muscle, a laminin antibody was used. The nuclei inside laminin include both myonuclei and satellite cells. The other nuclei were primarily from endothelial cells, fibroblasts or cells related to the immune system (Figure 5).

A small number of caspase-3 positive nuclei were observed in the control animals (Figure 5A), but there was a significant increase in apoptotic nuclei in the 9-14d group, including both intramuscular and interstitial cell nuclei (Figure 5D). There was no significant difference between the number of myonuclei per fiber between the different groups of rats, i.e., there was no net loss of nuclei over time. A striking finding was the increased number of enlarged nuclei in response to long-term immobilization and mechanical ventilation.

### Validation of array results with quantitative reverse transcription polymerase chain reaction (qRT-PCR)

In order to confirm array data, several up-regulated (*Murf1/Trim63*, *atrogin-1/Fbox32*, *Mybph*, *Map1lc3b*) and down-regulated (*MyHC-IIa*/*Myh2*, *Mybpc*) genes were selected. In addition, *Capn1 *whose expression levels did not change significantly was picked. Similar fold-change pattern were observed between the arrays and qRT-PCR (see Additional file 3), validating microarray results.

## Discussion

All critically ill ICU patients suffer from severe wasting and impaired muscle function, which delay respirator weaning and persist long after hospital discharge; thus, reducing quality of life. The present results demonstrate that the ICU muscle wasting represents unique muscle wasting condition where multiple signalling pathways are activated in a specific temporal pattern. In accordance with our previous observations, we found that most genes were turned on within 5 to 14 days, demonstrating body and muscle weight, muscle fiber size, single fiber contractile properties and contractile protein contents are maintained during the initial four days of the experimental ICU intervention [7].

The present results show: 1) A specific temporal pattern of protein degradation pathways with an early and maintained up-regulation of the atrogenes, followed by an activation of the autophagy-lysosome, calpain and ERAD protein degradation pathways at intermediate and long durations. 2) A dramatic down-regulation of a large number of sarcomeric proteins, including the molecular motor protein myosin, at intermediate and long durations. 3) Activation of protein synthesis, oxidative stress, several heat shock proteins/chaperones, cell cycle arrest and pro-apoptotic genes at intermediate and long durations. 4) Altered expression of genes involved in the regulation of muscle size at intermediate and long durations. 5) Activation of the caspase cascade at the longest duration. 6) Down-regulation of the caveolin-3, suggested to play a critical role in the altered mechanical signalling associated with the ICU intervention.

### Protein degradation

In concordance with many other atrophy models, there was an early (0.25-4d) and maintained (5-8d) 2-fold up-regulation of the muscle-specific E3 ligases, *atrogin-1/Fbox32 *and *Murf1/Trim63*, and a slightly decreased expression at the longest duration (9-14d). *Foxo1*, a member of the Foxo transcription factors, is one of several factors activating *Murf1 *and *atrogin-1*, but the *Foxo1 *activation of atrogenes was restricted to the early phase of the experimental ICU intervention (0.25-4d). NF-κB pathway is also involved in the induction of *Murf1 *[28]. Thus, after the initial Akt/Foxo activation, the up-regulation of the NF-κB pathway from 5 to 14 days is suggested to sustain the activation of *Murf1 *and protein degradation.

The autophagy-lysosome system was strongly up-regulated in response to the ICU intervention. *Cstl1 *was up-regulated more than 10-fold between 5 and 14 days. *Runx1 *and some cystatins (cathepsin inhibitors) were highly up-regulated from 5 to 14 days, presumably to preserve muscle mass from excessive autophagy [28], indicating that the autophagy-lysosome system is playing an important role in this type of muscle wasting especially at durations longer than 5 days, i.e., in accordance with disuse atrophy [29].

The calpain system, which disassembles myofibrillar proteins from the sarcomere to be ubiquitinated and degraded by UPS, was activated from 5 to 14 days. The expression of the muscle-specific isoform *Capn3 *decreased in response to the ICU intervention, i.e., in a similar way as previously reported during denervation and regeneration [30]. *Capn3 *is primarily involved in regulation and sarcomere remodelling [31] and does not contribute to the increased protein degradation.

The up-regulation of genes involved in ERAD represents a novel finding. ERADs target misfolded and unassembled secretory and transmembrane proteins from the endoplasmatic reticulum for degradation by the proteasome to control protein quality and maintain cell homeostasis [32,33].

### Sarcomeric proteins

Sarcomeric protein gene expression was significantly affected by the ICU intervention, contractile and regulatory proteins showed a dramatic down-regulation, primarily from 5 days and onwards. In contrast to other myosin and myosin-associated proteins, *Mybph *was up-regulated 3-fold at 9-14d. We have previously observed an increased *Mybph *expression in ICU patients with AQM during recovery [34]. The function of *Mybph *during atrophy and recovery is still unclear, but it may play an essential role in the organization, maintenance and reassembly of the thick filament.

### Protein synthesis and translation factors

The translational machinery was activated from 5 to 14 days in accordance with our previous observations showing an increased fractional protein synthesis rate at the longer durations in the experimental ICU model [7]. These results were concordant with the dramatic decline from day 9 to 14 of the synthesis repressor *Eif4ebp*; thus, promoting translation and protein synthesis at the latest phase of the intervention. This is in contrast to previous observations of an *Eif4ebp1 *up-regulation in response to hind limb unloading for 14 days in the rat [29] or human fibroblasts under microgravity [35]. Thus, removal of weight bearing alone appears to have a different effect compared with the effect of removal of both weight bearing and the internal strain during muscle contraction (mechanical silencing) associated with the ICU intervention.

### Oxidative stress response

Disuse and immobilization of skeletal muscles induce oxidative stress and reactive oxygen species (ROS) that activate apoptosis and proteolytic pathways [36,37]. In response to the oxidative stress under ICU condition, several antioxidant and chemoprotective genes were up-regulated.

### Apoptosis

From day 5 to 14, several cell cycle regulators, particularly those that promote cell cycle arrest that may lead to apoptosis [38], were up-regulated in response to the ICU intervention, and there was also an increase of several apoptotic genes and genes related to the p53 signalling pathway, as well as a late up-regulation (9-14d) of the caspase cascade. Thus, myonuclear apoptosis, as measured by the presence of cleaved caspase-3, was markedly elevated after 9 days of the ICU intervention, but not at earlier time-point. This is in contrast to observations of an early (12 h) increase in the number of apoptotic myonuclei in response to hind limb suspension 12 h [39]. This may also represent a fundamental difference in the atrophy process in models removing weight bearing alone compared with the complete mechanical silencing in response to the ICU intervention. When comparing the onset of myonuclear apoptosis with the onset of atrophy in the experimental ICU model, it becomes evident that the atrophy precedes apoptosis.

### Heat shock proteins/chaperones

A large number of chaperones that regulate cytoskeletal/sarcomere protein folding, assembly, protein degradation and protection against stress were up-regulated, in contrast to other atrophy models of disuse and denervation [29,40], but concordant with our previous observations in the porcine ICU model [8]. However, in the rat ICU model, up-regulation was significant at later durations (5-14d) than in piglets. This may reflect a developmental difference between the piglets, which were in an extremely fast growth phase, and the young adult rats, which had passed the rapid growth phase. *Hsp90 *binds to *Unc45b *regulating myosin folding and assembly [41]. In addition, both *Hsp90 *and *Hspb8 *are actively involved in chaperone-assisted degradation pathways [42]. The up-regulation of heat shock 105/110kDa protein (*Hsph1*) from 9 to 14 days paralleled the up-regulation of caspase-3 in accordance with the activation of *Hsph1 *by stress conditions such as oxidative stress or endoplasmic reticulum (ER) stress activating caspase-3 mediated apoptosis pathways [43]. *Hspb6 *expression, on the other hand, was down-regulated from 5 to 14 days and it may have a protective role against muscle atrophy [44].

### Skeletal muscle size regulators

Although some genes that promote muscle proliferation and growth such as fibroblast growth factors, *Jph1*, *Svil*, *Vgll2 *and *Ky *were down-regulated, a significant number of genes that stimulate muscle size were up-regulated, such as myogenic factors (*Myog*, *Myf5 *and *Myf6*). Further, the negative regulator of muscle growth, myostatin (*Mstn*), was down-regulated from 5 to 14 days at the same time as its inhibitor, follistatin (*Fst*) was up-regulated and the myostatin receptors (*Acvr2b *and *Acvr1*) were down-regulated. These results are in contrast with observations in other muscle atrophy models where myostatin is typically up-regulated [45-47] in line with the increased muscle mass observed in *Mstn^-/-^*, transgenic mice expressing a truncated form of activin type II receptor or high levels of follistatin and mice treated with a soluble form of activin type II receptor [48-51]. However, a similar down-regulation of myostatin was recently reported in ICU patients [52]. In addition, p38 MAPK and its downstream effector *Mk2 *have been reported to be up-regulated in atrophic condition, e.g., in patients with AQM [15], but they were found down-regulated in our ICU model. Myostatin has been proposed to activate p38 MAPK independently from Smad activation [53], thus indicating a possible mechanism for the inactivation of p38 pathway in this study as well as in critically ill ICU patients.

The muscle specific caveolin-3 (*Cav3*) plays an essential role in sarcolemma repair and mechanotransduction and it was down-regulated around 5-fold in our model. Defects in *Cav3 *are the cause of some neuromuscular diseases such as limb-girdle muscular dystrophy type 1C (LGMD1C) and rippling muscle disease (RMD). It was recently reported that down-regulation of *Cav3 *inhibits the myostatin pathway [54-56]. Thus, *Cav3 *is suggested to play a significant role in the altered intracellular signaling associated with the mechanical silencing in the ICU model and in the down-regulation of myostatin.

In addition, Jun B proto-oncogen (*JunB*), suggested to play a role in both the maintenance and hypertrophy of skeletal muscle mass [51], was up-regulated 2-fold after 9 days. This is different from the atrophy associated with fasting or denervation where *JunB *mRNA has been reported to be down-regulated [57,58]. Interestingly, earlier studies on *JunB *mRNA expression in denervated muscles have shown an up-regulation [59,60].

Taken together, these results indicate a stimulation of genes that regulate muscle development and growth at the longer durations (from 5 to 14 days). This is interpreted to reflect a compensatory mechanism to reduce the excessive down-regulation of sarcomeric proteins and up-regulation of different proteolytic pathways.

## Conclusion

To improve our understanding of the mechanisms underlying the muscle wasting/weakness and partial to complete loss of myosin in limb and trunk muscles in ICU patients with AQM, we conducted a time-resolved (6h to 14 d) gene expression analyses using a novel experimental rat ICU model. Novel temporal gene expression patterns have been uncovered, demonstrating the importance of time-resolved gene expression analyses to improve our understanding of the coordinated/complex mechanisms underlying the muscle wasting associated with the ICU intervention and providing new target genes and avenues for intervention studies.

## Methods

### Animals and tissue collection

A total of five sham operated controls and 23 experimental female Sprague-Dawley rats were included in this study, representing a subsample from a previous study in our laboratory [7]. The experimental rats were anaesthetized, treated with the neuromuscular blocker α-cobrotoxin and mechanically ventilated for durations varying from 6h to 4 days (n = 13), from 5 to 8 days (n = 4), and from 9 to 14 days (n = 6) (see Additional file 4). The experimental model has previously been described in detail [61,62]. Briefly, the following surgery and instrumentation was completed with sterile technique: 1) Precordial silver wire electrocardiogram (ECG) electrodes were implanted subcutaneously. 2) An aortic catheter (28-gauge Teflon) was inserted via the left carotid artery to record arterial blood pressure. 3) A 0.9-mm Renathane catheter was threaded into the left jugular vein to administer parental solutions. 4) Three subcutaneous EEG needle electrodes were placed into the skull above the right and left temporal lobes, and a third reference electrode was placed in the neck region. 5) Temperature was measured by a vaginal thermistor and servo-regulated at 37°C. 6) A silicone cannula was inserted in the urethra to continuously record urine output. The sham-operated control animals underwent the same interventions as the controls, but they were not pharmacologically paralyzed with alpha-cobratoxin. That is, sham operated-controls were anesthetized (isoflurane), spontaneously breathing, given intra-arterial and intra-venous solutions, and sacrificed within two hours after the initial anesthesia and surgery.

During surgery or any possible irritating manipulation, the anesthetic isoflurane level was at >1.5%, which maintained the following states: 1) the electroencephalogram (EEG) was synchronized and dominated by high-voltage slow-wave activity; 2) mean arterial pressure was 100 mmHg, the heart rate, 420 beats/min, and 3) there were no evident EEG, blood pressure or heart rate responses to surgical manipulation. Isoflurane was delivered into the inspiratory gas stream by a precision mass-flow controller. After the initial surgery, Isoflurane was gradually lowered (over 1-2 days) and maintained at <0.5% during the remaining experimental period. Rats were ventilated through a per os coaxial tracheal cannula at 72 breaths/min with an inspiratory and expiratory ratio of 1:2 and a minute volume of 180-200 ml and gas concentrations of 50% O_2_, 47% N_2_, and 3% CO_2_, delivered by a precision volumetric respirator. Intermittent respiratory hyperinflations (6 per hour at 15 cmH_2_O), positive end-expiratory pressure (1.5 cmH_2_O), and expiratory CO_2 _monitoring were continuous. Neuromuscular blockade (NMB) was induced on the first day (100 µg iv. α-cobrotoxin) and maintained by continuous infusion (250 µg/day, iv). Mechanical ventilation was initiated immediately after the NMB induction. Experiments were terminated at durations varying between 6 hours and 14 days. In no case did animals show any signs of infections or septicemia. The Ethical committee at Uppsala University approved all aspects of this study.

Gastrocnemius and plantaris muscles were dissected from the right leg immediately after euthanasia and were quickly frozen in liquid propane cooled by liquid nitrogen. The gastrocnemius muscle was divided into a proximal and distal part prior to freezing. Muscle samples were stored at -80°C for further analyses. The proximal part of the gastrocnemius was used for expression profiling and quantitative RT-PCR studies. The plantaris was used for immunofluorescence cytochemistry.

### Total RNA isolation and quantification

Total RNA was extracted from frozen gastrocnemius muscle (proximal part) tissue (10-30 mg) using a Qiagen RNeasy^® ^Mini Kit (Qiagen, Inc., Valencia, CA). Muscle tissue was homogenized using a rotor homogenizer (Eurostar Digital, IKAWerke). QIAshredder™ columns (Qiagen Inc., Valencia, CA) were used to disrupt DNA. Total RNA was eluted from RNeasy^® ^Mini columns with 30 μl of RNase-free water. RNA-concentrations were then quantified using the fluorescent nucleic acid stain, Ribogreen^® ^(Molecular Probes, Eugene, OR), on either a Chameleon PLC IIs (Hidex, Finland) fluorescence spectrophotometer. The same RNA preparation was used for expression profiling and quantitative RT-PCR.

### Expression profiling

Three micrograms of total RNA from the proximal gastrocnemius muscle samples were extracted and processed to generate biotin-labeled cRNA as previously described [63]. Each sample was then hybridized to Affymetrix Rat Gene 1.0 ST Array. The data discussed in this publication have been deposited in NCBIs Gene Expression Omnibus (GEO; [64]) and are accessible through GEO Series accession number GSE30848.

### Microarray data normalization and analyses

Subsequent analyses of the gene expression data was carried out in the freely available statistical computing language R using packages available from the Bioconductor project [65]. The raw data were normalized using the robust multi-array average [66] background-adjusted, normalized and log-transformed summarized values first suggested by Li and Wong in 2001 [67]. In order to search for the differentially expressed genes between the samples from the different days an empirical Bayes moderated t-test was applied [68], using the 'limma' package. A linear model was fitted to the data, control *vs*. 6 h-4 days, control *vs*. 5-8 days, control *vs*. 9-14 days. To address the problem with multiple testing, the *p*-values were adjusted according to Benjamini and Hochberg [69,70]. Probe sets with a minimum fold change of ± 2 (log_2 _ratio of ± 1) and adjusted *p*-value < 0.05 at least in one time point were included in further analyses. Each differentially expressed gene was flagged by Gene Ontology (GO) classification, including molecular function, biological process and cellular component [71] (a complete gene list is included in Additional file 2). The selected genes were grouped into a number of clusters according to similar expression patters with respect to time using *K*-means clustering analysis with Euclidian distance carried out with Genesis software [72]. Briefly, the *K*-means clustering algorithm initially divides genes into a number of equal sized groups based on a user-defined number (*k*). The centroid of each cluster is calculated as the average of the expression profiles. Each gene is then reassigned to the centroids that best match its expression pattern over time. Group centroids are then recalculated and the process is repeated until cluster composition converge [29,73]. In order to estimate the optimal number of clusters, Figure of Merit (FOM) analysis was done using Genesis software [72] (data not shown). The transcripts contained in each cluster were subjected to improved annotation using DAVID web based functional annotation tool [74]. Some of the functional categories were combined and some categorization was done manually, to improve the interpretative value of the data (see Additional file 1). PathwayExplorer software [75] was used to reconstruct the most relevant signalling pathways.

### Immunofluorescence cytochemistry

10 μm thick cross sections from the plantaris muscle were cut in a cryostat at -23 °C and put onto glass slides. The tissue sections were air dried at room temperature and stored at -80°C, for later analysis. The slides were then stained according to the following protocol: 1) Fixation: 2% PFA 15 min. 2) Washing: PBS 3 × 5 min. 3) Permeabilization: 0.1% Triton X-100. 4) Washing: PBS 5 min. 5) Blocking: 5% normal donkey serum and 3% horse serum 30 min. 6) Primary antibodies for cleaved caspase-3 (rabbit anti-human, Asp1751, Cell signaling, Danvers MA, 1:100), laminin (sheep anti-human, the binding site Birmingham UK, 1:1000) and MHC type I (1:13; mouse anti-rat, kindly provided by L.-E. Thornell, Umeå, University) were applied with a solution containing 5% normal donkey serum, 3% horse serum, 0.1% Triton X-100 and PBS. The sections were incubated at 4°C for 20 h. 7) Washing: PBS 5 × 3 min. 8) Secondary antibodies for cleaved caspase-3 (Alexa flour 488, donkey anti-rabbit, invitrogen Carlsbad CA, 1:1000), laminin (Alexa flour 633, donkey anti-sheep, invitrogen Carlsbad CA, 1:100) and anti MHC type 1 (Alexa flour 546, donkey anti-mouse, invitrogen Carlsbad CA, 1:1600) were applied with the same solution as with the primary antibody. The sections were incubated at 20°C for 60 min. 9) Washing: PBS 6 × 3 min, and 10) the sections were mounted in vectashield mounting medium with 4´,6-diamidino-2-phenylindole (DAPI). Sections were scanned in a Zeiss LSM 510 confocal microscope (Zeiss, Jena, Germany).

### Quantitative RT-PCR

qRT-PCR was used to quantify the mRNA levels for rat *Myh2 *(MyHC-IIa), *Mybpc*, *Mybph*, *atrogin-1/Fbox32*, *Murf1/Trim63*, *Capn1 *and *Map1lc3b *[GenBank:L13606, GenBank:X90475, GenBank:BC061993, GenBank:AY059628, GenBank:AY059627, GenBank:NM_019152 and GenBank:AY206669, respectively].

100 ng of total RNA from proximal gastrocnemius muscle samples were reverse transcribed to cDNA using Qscript cDNA supermix (Quanta Biosciences, USA). cDNA was amplified in triplicate using MyiQ™single color real time PCR detection system (Bio-Rad Laboratories, Inc., Hercules, CA, USA). The thermal cycling conditions include 95°C for 10 minutes, followed by 50 cycles of a two-step PCR with denaturation at 95°C for 15 sec and a combined annealing and extension step at 60°C for 1 min. Each reaction was performed in a 25µl volume with 0.4µM of each primer and 0.2µM probe. When optimising each PCR, the PCR products were run on 2% agarose gels to ensure that primer-dimer formation was not occurring. Taqman primers and probes were designed using the software Primer Express^® ^(Applied Biosystems, Foster City, CA, USA). Primer and probes sequences have been published elsewhere [7,9,76] and were purchased from Thermo Electron (Thermo Electron, Ulm, Germany). All primers and probes were purified by high-performance liquid chromatography. Threshold cycle (Ct) data obtained from running real-time RT-PCR was related to a standard curve to obtain the starting quantity (SQ) of the template cDNA, and the values were normalized against 18S rRNA [GenBank:AF102857].

### Statistics

For qRT-PCR and immunofluorescence cytochemistry, SigmaPlot software (Systat Software, Inc., CA, USA) was used to generate descriptive statistics. One way analysis of variance (ANOVA) was used to compare all groups. When the normality test failed, a one way ANOVA on ranks, i.e., Kruskal-Wallis one way ANOVA, was used. A Tukey's *post-hoc *contrast was performed to determine the means that were different at the significance level of *p *< 0.05; when normality failed, Dunn's *post-hoc *was used. Differences were considered significant at *p *< 0.05.

## Authors' contributions

MLD contributed to the tissue collection, performed RNA extraction, qRT-PCR tests, analyses of expression data, interpretations of results and prepared the manuscript. AMG controlled animals' intervention and collected the tissues. CO carried out the immunofluoresce cytochemistry. HG was responsible for the microarray hybridization, data normalization and statistical analyses. LL conceived the idea of using this approach, supervised the study, extracted the tissues and contributed to the writing of the manuscript. All authors have read and approved the final version of the manuscript.

## Supplementary Material

Additional file 1 ***K*-means clustering analysis**. List of the genes in each cluster and the functional categories associated to each cluster. Click here for file

Additional file 2 **Complete list of differentially expressed genes affected by ICU intervention**. List of 1583 significantly regulated genes at least in one time point with their corresponding fold change and adjusted ***p***-value at short (0.25-4d), intermediate (5-8d) and long (9-14d) duration, their GO classification and functional annotation. Click here for file

Additional file 3 **Results from qRT-PCR analyses**. Validation of microarray data by qRT-PCR. Correlation between fold changes from microarrays and from qRT-PCR. Click here for file

Additional file 4 **List with the time duration for each individual rat**. Click here for file
