# Supplementary material for: Muscle wasting and the temporal gene expression pattern in a novel rat intensive care unit model
Source: BMC Genomics. 2011 Dec 13;12:602. doi: 10.1186/1471-2164-12-602 (PMC3266306; doi:10.1186/1471-2164-12-602)
Supplement: Additional file 3 — Results from qRT-PCR analyses. Validation of microarray data by qRT-PCR. Correlation between fold changes from microarrays and from qRT-PCR. [file 1471-2164-12-602-S3.PDF]

Gene name	Gene symbol	Gene bank accession number	Method	FC (0.25-4d)	FC (5-8d)	FC (9-14d)
muscle ring-finger 1/tripartite motif-containing 63	*Murf1/Trim 63*	AY059627	Array	**2.8**	**3.4**	**2.2**
			qRT-PCR	**5.8**	**7.3**	**6.0**
atrogin-1/F-box protein 32	*Atrogin-1/Fbox32/ Mafbx*	AY059628	Array	**1.9**	**2.1**	**1.8**
			qRT-PCR	**4.6**	**5.4**	**4.0**
microtubule-associated protein 1 light chain 3	*Map1lc3b*	AY206669	Array	**2.1**	**2.7**	**2.3**
			qRT-PCR	**10.0**	**14.3**	8.1
myosin heavy chain 2a	*Myh2/MyHC-IIa*	L13606	Array	-2.5	**-10.9**	**-7.9**
			qRT-PCR	-1.52	**-13.5**	**-12.9**
myosin binding protein C	*Mybpc*	X90475	Array	-1.1	**-1.9**	**-4.2**
			qRT-PCR	-1.3	-1.9	-3.1
myosin binding protein H	*Mybph*	BC061993	Array	1.1	1.9	**3.0**
			qRT-PCR	-1.4	-1.2	1.4
calpain-1	*Capn1*	NM_019152	Array	-1.2	-1.3	-1.0
			qRT-PCR	4.5	4.4	2.4

FC = fold change at the three different time points (0.25-4d, 5-8d, 9-14d). Values in bold indicate differentially expressed genes after statistical analyses.

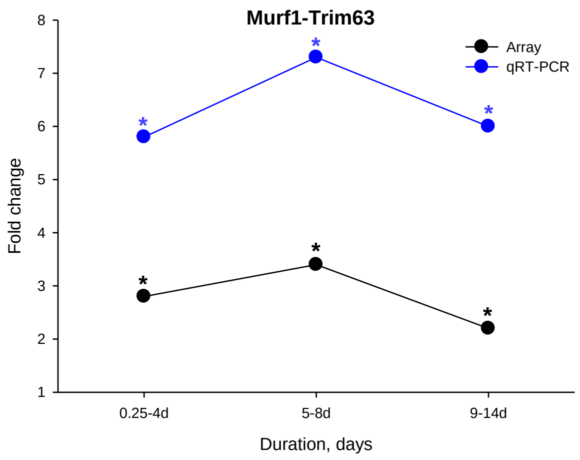

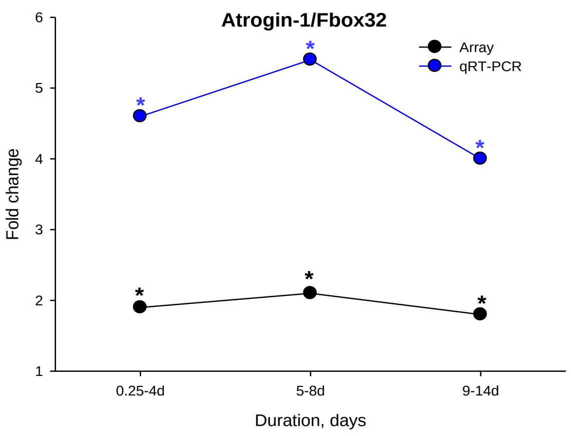

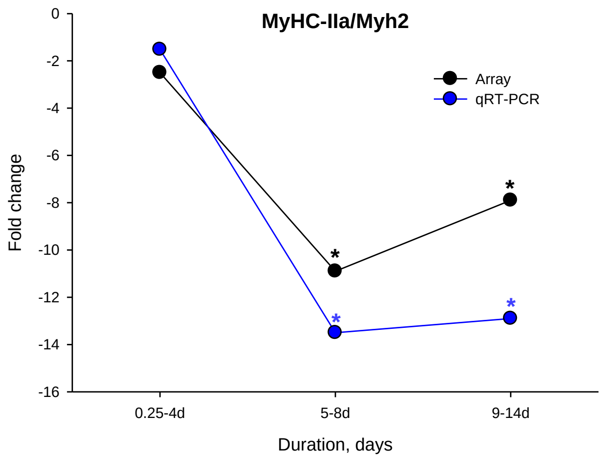

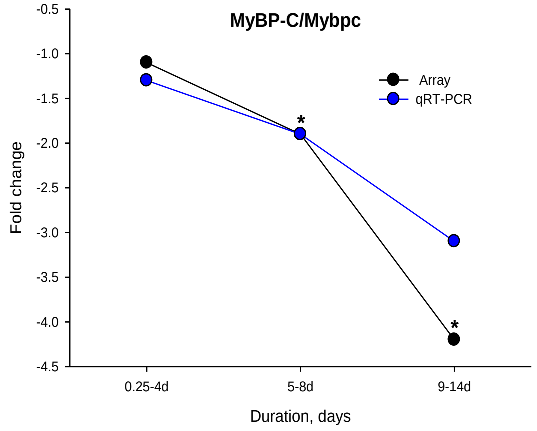

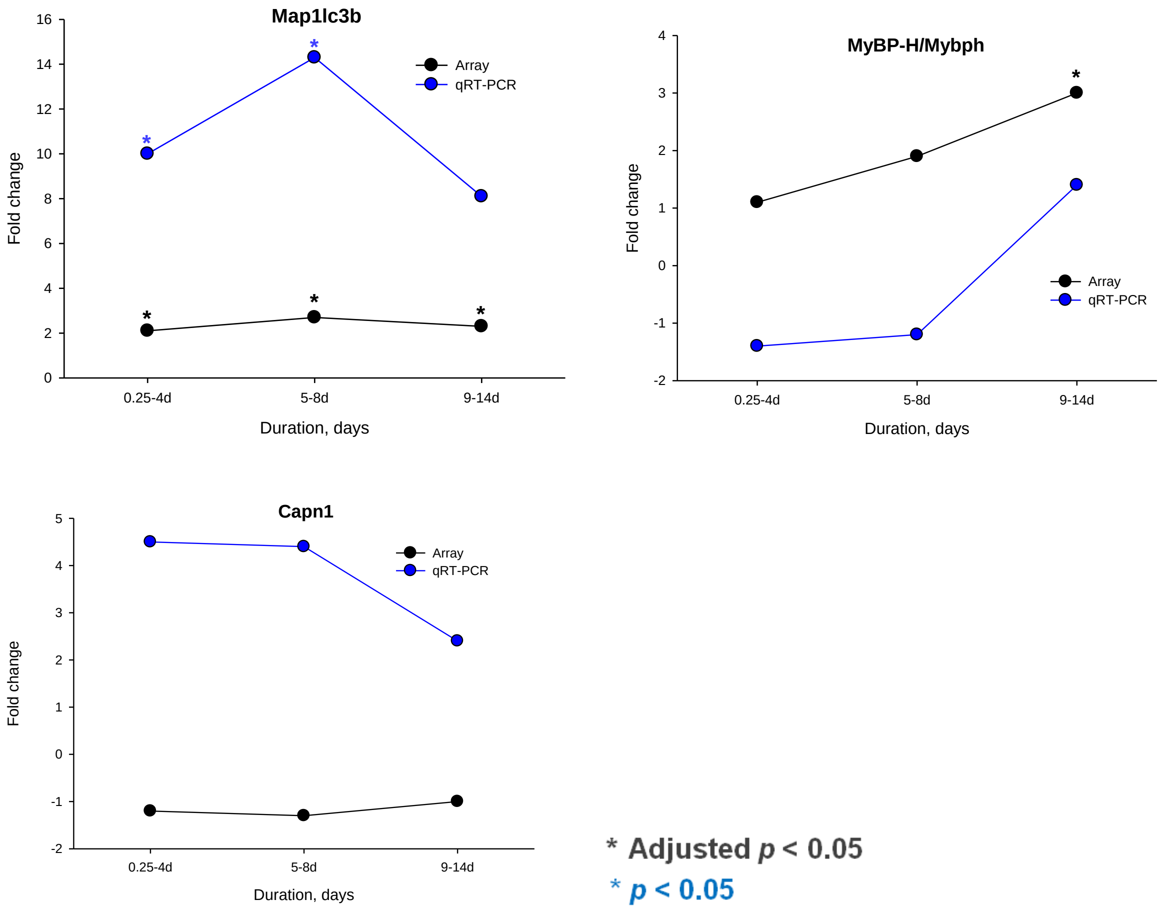

Map1lc3b
Fold change
16
14
12
10
8
6
4
2
0
0.25-4d
5-8d
9-14d
Duration, days
Array
qRT-PCR
*
MyBP-H/Mybph
Fold change
4
3
2
1
0
-1
-2
0.25-4d
5-8d
9-14d
Duration, days
Array
qRT-PCR
*
Capn1
Fold change
5
4
3
2
1
0
-1
-2
0.25-4d
5-8d
9-14d
Duration, days
Array
qRT-PCR
* Adjusted p < 0.05
* p < 0.05
